# Parental Information Needs and Intervention Preferences for Preventing Multiple Lifestyle Risk Behaviors Among Adolescents: Cross-sectional Survey Among Parents

**DOI:** 10.2196/42272

**Published:** 2023-04-04

**Authors:** Katrina E Champion, Emily Hunter, Lauren A Gardner, Louise K Thornton, Cath Chapman, Karrah McCann, Bonnie Spring, Tim Slade, Maree Teesson, Nicola C Newton

**Affiliations:** 1 The Matilda Centre for Research in Mental Health and Substance Use The University of Sydney Sydney Australia; 2 Department of Preventive Medicine, Feinberg School of Medicine Northwestern University Chicago, IL United States

**Keywords:** parents, adolescents, prevention, risk behaviors, intervention, mobile phone

## Abstract

**Background:**

Parents play an influential role in the health behaviors of their children, such as physical activity, dietary intake, sleep, screen time, and substance use. However, further research is needed to inform the development of more effective and engaging parent-based interventions targeting adolescent risk behaviors.

**Objective:**

This study aimed to assess parents’ knowledge about adolescent risk behaviors, barriers and facilitators to engaging in healthy behaviors, and preferences for a parent-based prevention intervention.

**Methods:**

An anonymous web-based survey was conducted from June 2022 to August 2022. Eligible participants were parents of children aged 11 to 18 years and were residing in Australia at the time of this study. The survey assessed the parents’ perceived and actual knowledge about Australian health guidelines for youth, parent and adolescent engagement in health behaviors, parenting style and attitudes, barriers and facilitators to engaging in healthy behaviors, and delivery and component preferences for a parent-based preventive intervention. Descriptive statistics and logistic regressions were conducted to analyze the data.

**Results:**

A total of 179 eligible participants completed the survey. The mean age of the parents was 42.22 (SD 7.03) years, and 63.1% (101/160) were female. Parent-reported sleep duration was high for both parents (mean 8.31, SD 1.00 hours) and adolescents (mean 9.18, SD 0.94 hours). However, the proportion of parents who reported that their child met the national recommendations for physical activity (5/149, 3.4%), vegetable intake (7/126, 5.6%), and weekend recreational screen time (7/130, 5.4%) was very low. Overall, parents’ perceived knowledge of health guidelines was moderate, ranging from 50.6% (80/158) for screen time to 72.8% (115/158) for sleep guidelines (for children aged 5-13 years). Actual knowledge was lowest for vegetable intake and physical activity, with only 44.2% (46/104) and 42% (31/74) of parents reporting correct guidelines for these behaviors, respectively. The key issues of concern reported by parents were excessive use of technology, mental health, e-cigarette use, and negative peer relationships. The top-rated delivery method for a parent-based intervention was via a website (53/129, 41.1%). The highest rated intervention component was opportunities for goal-setting (89/126, 70.7% rated very or extremely important), and other important program features were ease of use (89/122, 72.9%), paced learning (79/126, 62.7%), and appropriate program length (74/126, 58.8%).

**Conclusions:**

The findings suggest that such interventions should be brief and web based and should aim to increase parental knowledge of health guidelines; provide opportunities for skill-building, such as goal-setting; and include effective behavior change techniques, such as motivational interviewing and social support. This study will inform the development of future parent-based preventive interventions to prevent multiple lifestyle risk behaviors among adolescents.

## Introduction

### Background

Lifestyle risk behaviors, such as physical inactivity, poor sleep, sedentary behaviors (ie, sitting time and recreational screen time), poor diet, alcohol use, and tobacco smoking (the “Big 6”), are highly prevalent among adolescents worldwide. For example, an estimated 81% of adolescents globally report insufficient physical activity [1] and a recent meta-analysis of global adolescent dietary patterns found that 42.8% drank carbonated soft drinks at least once per day and 46.1% consumed fast food at least once per week [2]. The Big 6 are associated with a range of short- and long-term negative health outcomes, including obesity [3], mental illness [4,5], and chronic disease [6,7], which places considerable economic burden on already strained health care systems [8].

It is well established that adolescence is a critical period of behavior development, with lifestyle habits formed during this period typically persisting over time [9,10]. Thus, adolescence represents a valuable opportunity for the delivery of preventive interventions. In addition to being highly prevalent, lifestyle risk behaviors also tend to cooccur [11], with >80% of adolescents worldwide engaging in ≥2 risk behaviors concurrently and more than one-third engaging in ≥3 behaviors [12]. Engaging in multiple risk behaviors compounds the overall risk for chronic disease [13,14] and is associated with poor mental health outcomes [15]. Multiple health behavior change interventions seek to address this harmful trend, based on the notion that improving one behavior can increase self-efficacy to improve others without additional effort [16]. Although school-based multiple health behavior change interventions that are delivered via eHealth technology have proven effective in improving adolescent risk behaviors, such as physical inactivity and screen time, the effects are modest and short-lived [15]. Identifying intervention strategies that can augment the effects of school-based interventions are needed. Given the considerable influence of parents on adolescent health behaviors, involving parents in the preventive efforts represents an important opportunity to improve adolescent outcomes.

Parent-based interventions (ie, programs delivered through parents) provide the opportunity to continue intervention efforts within the home environment, the primary location of adolescent development [17]. Indeed, several modifiable parenting factors are associated with adolescent risk behaviors. For example, parental modeling is associated with increased diet quality [18], sleep quality [19], and physical activity [18] and decreased tobacco use [20], whereas rule-setting is associated with longer, better quality sleep [19] and decreased screen time [18,21]. Different parenting styles have also been linked to adolescent behaviors. For example, controlling parenting is associated with increased excessive screen time [21], and permissive parent behavior has been shown to increase sugar-sweetened beverage intake [22].

Despite this, surprisingly few interventions to prevent adolescent risk behaviors are parent based and family centered [23], and such interventions are typically characterized by high attrition [24] and low engagement [25], leading to decreased intervention success [26]. A recent meta-analysis of 36 randomized controlled trials [27] found that parent-based interventions for multiple lifestyle risk factors were associated with small improvements in adolescent physical activity and reduced screen time and junk food intake. However, parent-based interventions were not effective for alcohol and tobacco use, and no studies have addressed adolescent sleep. Further research is required to develop more effective parent-based interventions for preventing multiple adolescent risk behaviors. A critical part of this is working with parents to better understand their information needs, attitudes toward parenting and adolescent risk behaviors, and preferences for healthy lifestyle interventions.

### Objective

To inform the development of a new, parent-based intervention targeting the Big 6 lifestyle risk behaviors (physical inactivity, poor diet, recreational screen time, poor sleep, smoking, and alcohol use) among adolescents, this study aimed to assess parents’ knowledge about Australian health guidelines for the Big 6 for youth; explore attitudes and parenting practices in relation to the Big 6; and identify barriers, facilitators, and preferences for a parent-based preventive intervention.

## Methods

### Participants and Procedure

An anonymous cross-sectional survey was administered on the web through the Qualtrics (Qualtrics International Inc) survey platform from June 2022 to August 2022. Parents of children aged 11 to 18 years, who were residing in Australia at the time of this study, having access to a device connected to the internet to complete the survey were eligible. Participants were recruited through paid and unpaid advertising on social media (eg, Facebook and Twitter), as well as emails to professional and personal networks. Paid advertising was also conducted via state-based parents and carers associations, and hard copy flyers were distributed to households across Greater Sydney (Multimedia Appendix 1). These advertisements contained hyperlinks and QR codes to access the survey. All participants were required to provide informed consent before participation. Participants had the option upon completing the survey to enter a prize draw to win 1 of 5 gift vouchers valued at Aus $100 (US $68) each.

### Ethics Approval

This study was approved by the Human Research Ethics Committee of the University of Sydney (ethics approval: 2022/359).

### Measures

Demographic data included postcode, number and age of children, household income, education, employment, main language spoken at home, and marital status. Participants were also asked to self-report their age, sex, height, and weight (used to calculate BMI).

#### Parents’ Self-reported Health Behaviors

##### Physical Activity

To reflect the Australian physical activity guidelines for adults [28], the following two items were used to assess parents’ moderate to vigorous physical activity (MVPA): “About how many days per week do you do moderate intensity physical activity for at least 30 minutes?” and “About how many days per week do you do vigorous intensity physical activity for at least 20 minutes?” The response options ranged from 0 to 7 days per week.

##### Sedentary Time and Screen Time

Parents were asked the following two items: “In a typical day, how many hours do you spend sitting?” and “In a typical day, how many hours do you spend on your mobile phone, iPad, computer or another device for recreational (non-work) purposes?” (hours/day). The validity and reliability of single-item measures of daily sedentary time have been shown to be equivalent to those of other longer questionnaires [29].

##### Fruit and Vegetable Consumption

Average daily serves of fruits and vegetables were assessed using validated items [30,31].

##### Sleep Duration

Parents reported the time they usually went to bed at night and woke up each morning. Sleep duration (in hours) was calculated as the difference between wake time and bedtime [32]. Self-reported estimates of bedtime, wake time, and sleep duration have been shown to be reliable and valid [32].

##### Substance Use

Using items modified from the National Drug Strategy Household Survey [33], parents were asked to report how frequently they had consumed a standard drink of alcohol in the past 6 months (ranging from daily or almost daily to never); their smoking status (current smoker, former smoker, tried a couple of times, and never smoked); and their e-cigarette use (ever used and frequency of use). Self-reported substance use has been shown to be valid and reliable, particularly when participants are assured of confidentiality as they were in this study [34-36].

#### Parent-Reported Adolescent Health Behaviors

Parents were asked to select 1 adolescent (aged 11-18 years) child and answer the following questions in relation to that child.

##### Adolescent MVPA

A validated instrument [37] was used to assess adolescent MVPA. Parents were asked to report the number of days (0-7 days) their children were physically active for a total of at least 60 minutes per day over a typical week.

##### Recreational Screen Time

A modified version of the Adolescent Sedentary Activity Questionnaire [38] was used to assess recreational screen time (hours or minutes per typical school day and weekday). Sitting time was not assessed because of the lack of Australian guidelines for this risk behavior.

##### Fruit and Vegetable Consumption

Parents were asked to indicate how many serves of fruits and vegetables their adolescent children consume per day [30,31].

##### Sleep Duration

Parents were asked to estimate their children’s usual bedtime and wake time. Usual sleep duration per night (in hours) was calculated as the difference between wake time and bedtime. Similar items have been used in previous studies [32]; however, in this study, we did not differentiate between school days and weekend days.

##### Substance Use

A total of 3 items modified from the National Drug Strategy Household Survey [33] were used to assess adolescent use of alcohol, tobacco, and e-cigarettes (yes, no, sometimes, and unsure).

#### Knowledge About Adolescent Health Guidelines

A total of 7 items were used to assess parents’ perceived and actual knowledge about national health guidelines for adolescents in relation to physical activity, sleep duration (for children aged 5-13 years and 14-17 years), screen time, fruit and vegetable consumption, and alcohol use. National health guidelines provide a meaningful way to assess current health behaviors and are commonly used in adolescent health research, particularly in an intervention context [39-42]. Parents were first asked whether they knew the relevant guideline (yes, no, or unsure). For example, “Do you know the recommended amount of physical activity Australian teenagers should be getting?” Participants who responded “yes” were then asked to specify the recommended number of minutes or hours or serves for the relevant guideline.

#### Parenting Styles and Attitudes Toward Health Behaviors

The following two items, adapted from a study of parental support of child physical activity [43], were used to assess supportive parental attitudes: “I believe it is important that my child develops healthy lifestyle behaviours (physical activity, healthy diet, and healthy sleep)” and “Supporting my child’s participation (through driving, participating, or paying for their activities, etc.) in physical activity is important to me.”

A total of 3 items were adapted from Theory of Planned Behavior questionnaires, which evaluate perceived norms, perceived behavioral controls, and intentions [44], including “I consistently uphold expectations for healthy behaviour and openly communicate these expectations with my child in a positive and supportive manner.”

One item, “I am able to do the things that will improve my child’s behaviour” was taken from the Brief Parent Self-Efficacy Scale [45], a validated measure to assess parent beliefs of self-efficacy and capability. Responses were coded from 1 (strongly disagree) to 5 (strongly agree).

#### Parental Control and Rule-Setting

A total of 6 items adapted from the Parenting Strategies for Eating and Activity Scale [46] were used to assess parental control and rule-setting in relation to each of the Big 6, including *“*I monitor and set rules for my adolescent child in relation to how much screen time they can have per day.” Responses were coded as 0 (never and rarely) or 1 (sometimes, often, and always).

#### Perceived Importance of Health Behaviors

An open-ended question was used to ask parents what they perceived to be the biggest issues and concerns for young people today. Participants were also asked to rank which of the Big 6 items they would most like to support their adolescent child to improve, change, or resist (1=most important to 6=least important). Parents were also asked a similar question regarding their own behaviors.

#### Barriers and Facilitators

Parents were asked about the barriers and facilitators they faced and those that their child faced in engaging in healthy behaviors. For example, “What are the main things that stop YOU from engaging in healthy behaviours” and “What would you consider to be the biggest factor or factors that allow YOUR ADOLESCENT CHILD to engage in health behaviours?*”* Items were adapted from a systematic review of barriers to and facilitators for the uptake and maintenance of healthy behaviors [47].

#### Intervention Preferences

Parents who indicated that they would be interested in accessing a healthy lifestyle program about adolescent health were asked about their intervention preferences (eg, frequency, format, length, content, and components). A combination of open-ended questions, such as *“*Is there anything that would encourage you to participate in a parent-based healthy lifestyles program”; multiple choice questions, such as “How much time would you be willing to spend per week completing the program”; and Likert scales, including one for “How important is an easy-to-use parenting program to you,” were used. Items were adapted from a previous study of parents to inform the development of an eHealth alcohol prevention program [48].

### Statistical Analysis

Descriptive statistics were conducted using SPSS (version 26; IBM Corp) to describe the demographic data (frequency, percentage, mean, SD, and range). Scores on validated measures were calculated using established scoring procedures, and the mean responses and percentages were reported. Bivariate correlations were conducted to examine the associations between parents’ engagement in the 6 risk behaviors and parent-reported youth engagement in the risk behaviors. Chi-square tests were conducted to explore differences in parental rule-setting by adolescent age (children aged 11-14 years vs those aged 15-18 years) for each risk behavior. Binary logistic regression was conducted to examine the associations between parental knowledge of national guidelines for each risk behavior and their child’s adherence to the guidelines. Separate logistic regression models were conducted to examine whether parental rule-setting about the Big 6 was associated with adolescent engagement in each risk behavior (ie, meeting guidelines, yes or no). Owing to the small sample size and multiple tests conducted, a conservative *P* value was used (*P*<.01). Thematic analysis was used to examine the open-ended responses. Using an inductive approach [49], 1 author (EH) coded the responses, examined the data for frequent or significant responses, and grouped them according to key themes. A second author (KEC) confirmed the coding and grouping, with any discrepancies resolved via discussion.

## Results

### Sample Characteristics

A total of 353 participants completed the survey. Of them, 49.3% (174/353) were excluded per eligibility criteria (19 individuals had no children; 93 parents did not have children aged between 11 and 18 years; 4 parents were aged <24 years; and 58 individuals were not living in Australia), resulting in 50.7% (179/353) eligible participants. The mean age of the parents was 42.22 (SD 7.03) years, and 63.1% (101/160) were female. The mean age of the selected index child was 14.62 (SD 1.81; range 11-18) years. Table 1 presents the characteristics of the entire sample.

**Table 1:** Sample characteristics (N=179).^a^

			Values, n (%)
**Parent sex (n=160)**			
	Female	101 (63.1)	
	Male	57 (35.6)	
	Prefer not to answer	2 (1.2)	
**Household income, Aus $^b^ (n=161)**			
	<18,200	3 (1.9)	
	18,201-45,000	19 (11.8)	
	45,001-120,000	52 (32.3)	
	120,001-180,000	48 (29.8)	
	>180,001	33 (20.5)	
	Prefer not to say	6 (3.7)	
**Education (n=161)**			
	Postgraduate degree	49 (30.4)	
	Graduate diploma or graduate certificate	20 (12.4)	
	Bachelor’s degree	57 (35.4)	
	Advanced diploma or diploma	17 (10.6)	
	Certificate I, II, III, or IV	12 (7.4)	
	High school	6 (3.7)	
**Employment (n=160)**			
	Employed full-time	115 (71.9)	
	Employed part-time or casual	31 (19.3)	
	Unemployed	2 (1.2)	
	Retired	1 (0.6)	
	Home duties	8 (5)	
	Other	3 (1.9)	
**Main language spoken (n=179)**			
	English	146 (96.7)	
	Other	5 (2.8)	
**Postcode (n=121)**			
	Living in NSW^c^	71 (58.7)	
**Marital status (n=160)**			
	Married	125 (78.1)	
	Divorced	12 (7.5)	
	De facto	12 (7.5)	
	Other	11 (6.9)	
**Age of adolescent** **(index child), years** **(n=132)**			
	11	5 (3.8)	
	12	12 (9.1)	
	13	23 (17.4)	
	14	22 (16.7)	
	15	26 (19.7)	
	16	21 (16)	
	17	16 (12.1)	
	18	7 (5.3)	

^a^Discrepancies in participant N values are due to missing data.

^b^Aus $1=US $0.68 (at time of prize draw).

^c^NSW: New South Wales.

### Parent and Adolescent Health

Most parents rated their own health as “good” (67/157, 42.7%); however, 46.4% (83/179) reported having been diagnosed with a chronic condition, most commonly arthritis (24/179, 13.4%) and high blood pressure (21/179, 11.7%). On average, parents were overweight (BMI: mean 25.75, SD 9.12). Table 2 reports the parent-reported engagement in each Big 6 risk behavior for parents and adolescents. The parents reported consuming an average of 1.6 serves of fruit per day, but only 2.4 serves of vegetables, well below the national recommendation of 5 daily serves (Multimedia Appendix 1 lists the Australian guidelines for each behavior). However, on average, sleep duration was adequate among the sample, with parents sleeping for an average of 8.3 (SD 1.38) hours per night (range 5-12.5 hours). Although only 19.7% (31/157) were current tobacco smokers, nearly one-quarter of the parents (37/156, 23.7%) reported having used e-cigarettes, of whom 51% (19/37) reported vaping at least weekly.

**Table 2:** Parent and adolescent engagement in Big 6 risk behaviors.

				Parent’s health behaviors		Adolescent health behaviors
Fruit intake (serves/day), mean (SD)				1.66 (0.98)		1.84 (1.09)
Vegetable intake (serves/day), mean (SD)				2.37 (1.33)		2.14 (1.18)
**Physical activity (days per week), mean (SD)**						
	30 minutes moderate			4.13 (1.84)		N/A^a^
	20 minutes vigorous			3.07 (1.92)		N/A
	60 minutes MVPA^b^			N/A		3.93 (1.6)
Sleep duration (hours/day), mean (SD)				8.31 (1.00)		9.18 (0.94)
Sitting time (hours/day), mean (SD)				7.15 (3.33)		N/A
**Recreational screen time (hours/day), mean (SD)**				2.77 (1.51)		N/A
	Weekday			N/A		2.42 (1.55)
	Weekend			N/A		4.48 (2.47)
**Alcohol use, n (%)**						
	**Ever used^c^**					
		Yes	N/A		9 (5.8)	
		No	N/A		99 (63.5)	
		Sip or 2	N/A		40 (25.6)	
		Unsure	N/A		8 (5.1)	
	**Frequency^d^**					
		Daily or almost daily	21 (13)		N/A	
		Weekly	40 (24.7)		1 (11.1)	
		2-3 times monthly	27 (16.7)		7 (77.8)	
		Once a month	21 (13)		1 (11.1)	
		Less than a month	32 (19.8)		N/A	
		Never	21 (13)		N/A	
**Tobacco use^e^, n (%)**						
	Never smoked			67 (42.7)		N/A
	Tried smoking			26 (16.6)		N/A
	Former smoker			33 (21)		N/A
	Current smoker			31 (19.7)		N/A
	**Ever used^f^**					
		Yes	N/A		12 (7.8)	
		No	N/A		125 (81.7)	
		Unsure	N/A		16 (10.5)	
	**Frequency^g^**					
		<Monthly	N/A		6 (50)	
		Once a month	N/A		1 (8.3)	
		2-3 times a month	N/A		1 (8.3)	
		Weekly	N/A		3 (25)	
		Daily or almost daily	N/A		1 (8.3)	
**e-Cigarette use, n (%)**						
	**Ever used^h^**			37 (23.7)		N/A
		Yes	N/A		24 (15)	
		No	N/A		117 (73.1)	
		Unsure	N/A		19 (11.9)	
	**Frequency^i^**					
		<Monthly	9 (24.3)		4 (17.4)	
		Once a month	4 (10.8)		3 (13)	
		2-3 times monthly	5 (13.5)		11 (47.8)	
		Weekly	9 (24.3)		1 (4.3)	
		Daily or almost daily	10 (27)		4 (17.4)	

^a^N/A: not applicable.

^b^MVPA: moderate to vigorous physical activity.

^c^Adolescents: n=156.

^d^Parents: n=162; adolescents: n=9.

^e^Parents: n=157.

^f^Adolescents: n=153.

^g^Adolescents: n=12.

^h^Parents: n=156; adolescents: n=160.

^i^Parents: n=37; adolescents: n=23.

Approximately half of the parents (71/131, 54.2%) reported that their child consumed the recommended minimum of 2 serves of fruit per day; however, only 5.6% (7/126) indicated that their child ate the recommended 5 serves of vegetables. Similarly, only 3.4% (5/149) of parents reported that their adolescent child achieved the recommended guideline of 60 minutes of MVPA per day. In contrast, adolescent sleep duration was perceived to be high, with parents reporting a mean of 9.18 (SD 1.58) hours per night and the vast majority perceiving their child to meet recommended sleep guidelines (33/37, 89% for children aged <14 years; 68/81, 84% for children aged >14 years). A total of 39.2% (51/130) of parents indicated that their adolescent child met the national guidelines of <2 hours of daily screen time on school days; however, only 5.4% (7/130) of parents reported that their child met the guidelines on weekend days. In terms of substance use, 5.8% (9/156) of parents reported that their child had ever used alcohol; 7.8% (12/153) of parents reported that their children had smoked tobacco, and 15% (24/160) of the parents reported that their children had ever used e-cigarettes. Parent vegetable intake was moderately and positively correlated with parent-reported adolescent vegetable intake (*r*=0.618; *P*<.001), however, the correlations for all other risk behaviors were weak (Multimedia Appendix 1).

### Knowledge About Adolescent Health Guidelines

**Figure 1:**
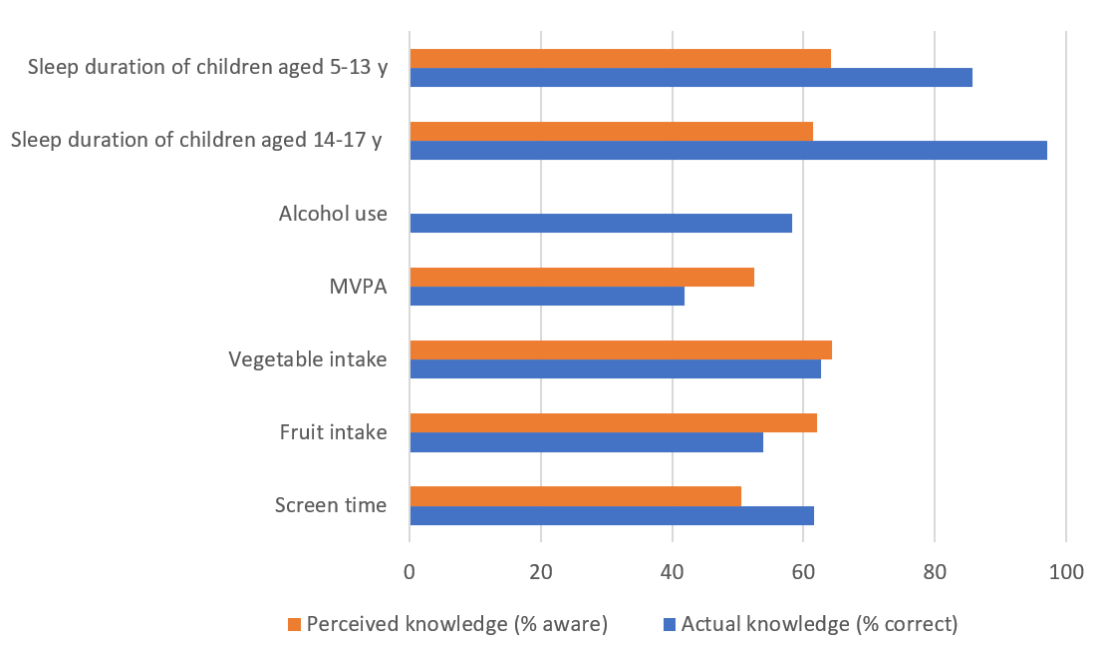
Parent knowledge of national youth guidelines for the Big 6 (perceived knowledge not assessed for alcohol use). MVPA: moderate to vigorous physical activity.

Figure 1 depicts parents’ perceived (% aware) and actual knowledge (% correct) of national health guidelines for each risk behavior. Overall, perceived knowledge was moderate, ranging from 50.6% (80/158) for screen time to 72.8% (115/158) for sleep guidelines (for children aged 5-13 years).

Parents tended to overestimate their knowledge regarding fruit and vegetable intake and MVPA. For example, although nearly two-thirds of the parents (111/157, 70.7%) thought that they knew how many serves of fruit their child should be consuming, only half of them (56/104, 53.8%) reported the correct guideline. Of the parents who answered incorrectly, most overestimated the recommended daily serves (45/104, 43.2%, thought the guideline was ≥3 serves). Actual knowledge was lowest for vegetable intake and MVPA, with only 44.2% (46/104) and 42% (31/74) of parents reporting the correct guidelines for these behaviors, respectively. Just over half of the sample (92/158, 58.2%) knew that it is recommended that adolescents must not consume alcohol, and 60% (45/73) of the parents correctly identified that the recommended daily limit for screen time was 2 hours.

In contrast, actual knowledge of sleep guidelines was very high. A total of 85.6% (90/105) of the parents correctly reported that 9 to 11 hours per night is the recommended guideline for younger children. Similarly, although 70.1% (110/157) of parents reported an awareness of the guidelines for children aged 14 to 17 years, nearly all the parents (100/103, 97.1%) correctly identified the recommended 8 to 10 hours per day. Results from the logistic regression analyses indicated that except for screen time on school days, parental knowledge of national health guidelines was not significantly associated with adolescents’ adherence to the guidelines (Multimedia Appendix 1).

### Parenting Styles, Attitudes, and Rule-Setting

Most parents reported supportive and positive attitudes toward parenting and their child’s health. Many parents (120/149, 80.6%) agreed that supporting their children’s participation in healthy behaviors was important to them, and most of them (126/147, 85.7%) reported that they enjoy helping their children engage in physical activity, for example, by driving their child to a sports practice or watching their child participate in activities. In terms of role-modeling, most parents (123/149, 82.5%) agreed that they model healthy lifestyle behaviors for their adolescent children, and nearly all (137/150, 91.3%) reported that they upheld expectations for their child and positively communicated them. Finally, most participants reported a strong sense of parenting self-efficacy, with 71.2% (104/146) agreeing that they could do the things that would improve their child’s behavior.

Parental control and rule-setting were moderate to high across the 6 risk behaviors. Nearly three-quarters of the parents (104/145, 71.7%) reported that they monitored and set rules about their child’s bedtime and wake time. Approximately half of the parents indicated that they monitored what their child could and could not eat or drink (83/148, 56.1%), how much time they could spend on screens per day (81/146, 55.4%), and how much physical activity they engaged in (75/147, 51%). In terms of rules about substance use, 67.9% (99/146) and 67.3% (99/147) of the participants reported monitoring and setting rules about alcohol use and tobacco use, respectively. Nearly all parents (57/62, 92%) reported setting rules about alcohol use for younger adolescents (aged 11-14 years), compared with three-quarters of parents (47/63, 75%) of older adolescents (15-18 years; *P*=.01). Similarly, parental rule-setting regarding tobacco use was higher for younger adolescents (55/62, 86%) compared with older adolescents (45/63, 71%); however, this difference was not statistically significant (*P*=.02). Except for rules about dietary intake, the proportion of parents setting rules for physical activity, screen time, and sleep was higher for younger than older adolescents; however, these differences were not significant (Multimedia Appendix 1). Results from the logistic regression analyses indicated that across all the Big 6, parental rule-setting was not significantly associated with adolescents’ engagement in the risk behaviors (Multimedia Appendix 1).

### Perceived Importance of Health Behaviors

When asked about the biggest issue facing young people today, parents commonly reported concerns about adolescents’ use of technology, including addiction to smartphone devices and social media. Other concerns included mental health, substance use, and negative peer relationships. The key themes extracted from the open-ended responses of 62 participants are presented in Table 3. A total of 36% (54/150) of parents ranked diet as the behavior they would most like to support their adolescent child to change or improve, whereas 17.3% (26/150) of parents ranked screen time as the most important, and 15.3% (123/150) of parents selected sleep. Similarly, when thinking about their own behaviors, 41.5% (61/147) of parents ranked diet as the most important behavior to change, followed by physical activity (26/147, 17.7%), and sleep (19/147, 12.9%).

**Table 3:** Summary of key themes in relation to parents’ biggest concern for adolescents today.

Theme			Example
**Technology**			
	Excessive screen time	“Addicted to electronic devices” (Female parent of a child aged 17 years)	
	Social media	“The content (not quantity) that they are exposed to online” (Female parent of a child aged 12 years)	
	Video games	“Staying up late playing games” (Female parent of a child aged 16 years)	
**Mental health**			
	Mental health	“Vaping” (Female parent of a child aged 13 years)	
	Stress	“Stress from school” (Female parent of a child aged 17 years)	
	Substance use	“Social isolation and mental health” (Female parent of a child aged 12 years)	
**Peer relationships**			
	Peer pressure	“Social pressures to conform” (Female parent of a child aged 16 years)	
	Peer acceptance	“Being accepted by their peers” (Female parent of a child aged 14 years)	
**Physical health**			
	Insufficiently active	“Low activity levels” (Male parent of a child aged 15 years)	

### Barriers and Facilitators to Engaging in Health Behaviors

Table 4 presents the top 3 barriers, motivators, and enablers related to parents’ own health behaviors and their adolescent children’s behaviors. The most common barrier for both parents and adolescents was lack of motivation, endorsed by 42.5% (76/179) and 50.8% (91/179) of the parents, respectively. In terms of motivating factors, the most endorsed option for parents was setting a good example for their children (108/179, 60.3%) and enjoying a healthy lifestyle for adolescents (87/179, 48.6%). The top 3 factors that enabled a healthy lifestyle were the same for parents and their adolescent children, namely, integration of healthy behaviors into their lifestyle and routine, access to healthy options, and social support and encouragement (Table 4).

**Table 4:** Barriers, motivators, and enablers associated with healthy behaviors (N=179).

		Parents, n (%)	Adolescents, n (%)
**Barriers**			
	Lack of motivation	76 (42.4)	91 (50.8)
	Limited facilities or resources	43 (24)	N/A^a^
	Financial constraints	41 (23)	N/A
	Lack of access to healthy options in the home	N/A	39 (21.8)
	Lack of time	N/A	34 (19)
**Enablers**			
	Integration of healthy behaviors into lifestyle	111 (62)	93 (52)
	Access to healthy options	82 (45.8)	81 (45.2)
	Social support and encouragement	59 (33)	95 (53.1)
**Motivators**			
	Setting a good example	108 (60.3)	N/A
	Desire to be healthy	103 (57.5)	69 (38.5)
	Mental health benefits	100 (55.9)	71 (49.7)
	Enjoyment of a healthy lifestyle	N/A	87 (48.6)

^a^N/A: not applicable.

### Intervention Preferences

A minority of participants (21/156, 13.5%) indicated that they were not interested in accessing a parent-based healthy lifestyles intervention to improve adolescent health owing to following reasons: they already had adequate knowledge, had already implemented health-promoting strategies at home, and reported a lack of time. Of the nearly three-quarters of parents (115/156, 73.7%) who indicated that they would be interested, 41.1% (53/129) of the parents said their preferred delivery method was through a website. The second most preferred delivery method was face-to-face sessions (29/129, 22.5%), followed by mobile apps (25/129, 19.4%). Most parents (80/134, 67.1%) indicated that they would be willing to spend 10 to 40 minutes per week on a parent-based program, with approximately one-quarter of the parents (32/134, 23.9%) reporting that they would spend 30 to 40 minutes. Regarding intervention components and content, most parents (89/126, 70.7%) rated opportunities for goal-setting as “very” or “extremely” important for developing a parent-based adolescent health intervention, followed by parent-child joint sessions (88/125, 70.4%) and case studies (81/123, 65.9%). Other important program features identified by the parents were ease of use (89/122, 72.9%), appropriate program length (74/126, 58.8%), and paced learning (79/126, 62.7%).

## Discussion

### Principal Findings

This study aimed to explore parents’ knowledge about the Australian guidelines for the Big 6 risk factors (physical inactivity, poor diet, recreational screen time, poor sleep, smoking, and alcohol use); attitudes and parenting practices in relation to the Big 6; barriers and facilitators; and preferences for a parent-based preventive intervention. Although most parents perceived themselves and their adolescent children to be achieving adequate sleep, engagement in the other lifestyle risk behaviors was high. Parents reported low vegetable consumption by themselves and their adolescent child, which aligns with the latest national prevalence data on youth [50]. Although closer to the recommended serves, the average fruit consumption was still below the national guideline for both parents and adolescents. Perhaps unsurprisingly, when asked which of the Big 6 behaviors they would most like to change, parents reported dietary habits as the most common response for both themselves and adolescents. In terms of adolescents, the most concerning behaviors were the low proportion of youth meeting the national recommendations for MVPA and screen time on weekends. This is consistent with the high rates of these risk behaviors observed in other large samples of adolescents in Australia [9] and worldwide [1,51] and highlights the urgent need for preventive measures to improve these behaviors among youth.

Overall, parental self-efficacy was high, and most parents reported a supportive parenting style and positive attitudes toward their child’s health behaviors. However, parental knowledge of the national youth guidelines for the Big 6 was moderate. Except for sleep, there were clear knowledge gaps regarding the guidelines for fruit and vegetable intake, MVPA, alcohol use, and screen time, as well as mismatches between perceived and actual knowledge of the guidelines. Concerningly, a large proportion of parents (66/158, 41.8%) were unaware that abstaining from alcohol was the safest option for adolescents, as stipulated in the national guidelines, highlighting a clear priority for further parental education. Parental control and rule-setting were moderate to high across the 6 risk behaviors, with parents more likely to set rules about alcohol and tobacco use for younger adolescents (aged 11-14 years) than for older adolescents (aged 15-18 years); however, these were not statistically significant differences. This aligns with research suggesting that parental rule-setting should be appropriate to an adolescent’s developmental stage to effectively protect against risk behaviors [52]. Although higher parental behavioral control, or rule-setting, may be suitable for younger adolescents, there is an increased need for autonomy and independent decision-making regarding health behaviors alongside rules as adolescents grow [52,53]. However, given the strong evidence for rule-setting as a protective factor against adolescent alcohol and tobacco use and related harms [54,55], parents should set rules in line with the Australian National Guidelines for alcohol and tobacco use, supporting abstinence for those aged <18 years. Although parental rule-setting was not significantly associated with adolescents’ engagement in any of the Big 6 risk behaviors, prior research has found rule-setting to be important for adolescent behaviors, particularly in relation to alcohol use [54,55], sleep [19], and screen time [56]. This suggests that rule-setting should be considered when designing new parent-based interventions to improve adolescent health, and further research exploring associations between the Big 6 risk behaviors and parental control and rule-setting among larger samples of parents and youth may be warranted.

### Comparison With Prior Work

There is some evidence to suggest that adolescent knowledge of guidelines is associated with better adherence to guidelines [57]; however, the relationships between parental knowledge of guidelines and adolescent health behaviors have not been well studied. Prior research has found that the related concept of parental health literacy, that is, competency in accessing, understanding, appraising, and applying health-related information [58], is associated with adolescent health behaviors, particularly in relation to healthy dietary intake [59]. For example, in a German study of >4000 parents, children (>11 years) of parents with better health literacy reported eating more vegetables and salad and consuming fewer sweetened beverages [59]. In another study of parent-adolescent dyads in the United States, the odds of obesity among youth decreased with higher levels of parental health literacy [60]. Although we did not find evidence of an association between parental knowledge of national health guidelines and adolescents’ adherence to guidelines in this study, our analyses were limited by our small sample size, low cell counts for some variables, and parent-reported, rather than adolescent-reported, risk behaviors. Nonetheless, in the context of prior work, the findings suggest that health promotion campaigns and preventive interventions are needed to improve parents’ health literacy and knowledge about national guidelines for the Big 6 risk behaviors, particularly in relation to diet, MVPA, alcohol use, and screen time. However, increasing knowledge alone is unlikely to be sufficient to promote behavior change among adolescents [61], and including opportunities for skill-building for parents and adolescents is also critical.

### Implications for Prevention

This study has several important implications for parent-based interventions to prevent multiple lifestyle risk behaviors among adolescents. First, the most highly rated intervention component by parents was goal-setting, a commonly used and effective behavior change technique (BCT) in interventions targeting the Big 6 risk factors [62]. This suggests that future interventions should include opportunities for parents to set and track goals to improve their families’ health. In addition, our finding that the most common barrier to engaging in healthy behaviors was a lack of motivation suggests that future parent-based interventions should include motivational interviewing or other effective BCTs such as “feedback on behaviour,” which has been shown to increase engagement and provide motivation [63]. Other common barriers reported by parents were limited facilities or resources and financial constraints, indicating that interventions should seek to educate and empower parents about adopting healthy behaviors, such as eating healthily on a budget and doing physical activity beyond organized sports and paid activities, which are within budget and practical limitations. Similar research has found that practical constraints such as cost and access to resources play an important role in parents’ engagement in adolescent healthy lifestyle programs [64]. In terms of enabling factors, the top 3 factors were integration of healthy behaviors into lifestyle, access to healthy options, and social support and encouragement. Indeed, social support is increasingly recognized as an important BCT for health interventions targeting multiple behaviors [65,66] and was identified as the most effective BCT in a recent systematic review of parent-based interventions [27]. Therefore, future prevention efforts for parents should aim to support families in embedding new skills into their lifestyle and routines and provide opportunities for social support, both emotional and practical.

The findings from this study also support the use of eHealth approaches, particularly web-based interventions. eHealth programs can be completed remotely and flexibly and reduce financial, travel, and work-related costs, making them a viable option for many parents [67]. This includes families from disadvantaged backgrounds who typically have higher rates of chronic disease risk factors [68] and poorer health outcomes [69,70]. In addition, other findings suggest that future parent-based interventions targeting the Big 6 should be brief (10-40 minutes per week), easy to use, and offer paced learning. Finally, qualitative data indicated that parents were primarily concerned about adolescents’ use of technology, including the excessive use of smartphones, social media, and gaming, as well as mental health, substance use, and negative peer relationships. This coincides with recent data indicating the growing prevalence of screen time [9], vaping [33], and poor mental health [71] among Australian adolescents, particularly in the aftermath of the COVID-19 pandemic [72,73]. Previous research has similarly advocated the inclusion of parents within adolescent interventions, particularly those targeting mental health [64,74], as a means of maximizing outcomes. Taken together, this suggests that parents are likely to be receptive to a brief eHealth program designed to address contemporary issues, such as vaping and mental health, alongside programs that target chronic disease risk factors and those that aim to circumvent the practical (eg, transport [74], cost [64,74], scheduling [74]) and social (eg, stigma [74] and fear of judgment about parenting) barriers that have traditionally hindered engagement.

### Limitations

This study has several limitations that should be considered. First, although 353 parents responded to the survey, nearly half of them (n=174, 49.3%) were deemed ineligible, resulting in a small sample of 179 parents. In addition, although we recruited participants nationwide via social media, most participants in the sample were well educated, employed, and English speaking, which limits the generalizability of our results. Given the well-known health inequalities among parents and adolescents from low socioeconomic backgrounds and culturally and linguistically diverse populations, further work among more diverse populations is needed. In contrast, nearly 40% of our sample were male participants, which is a strength, as fathers typically find it difficult to engage in this type of prevention-related research [48]. In addition, this study was cross-sectional in nature, and further longitudinal research is needed. Another limitation is that parents were asked to self-report their engagement in risk behaviors, increasing the probability of bias and random error. Self-reporting is subject to several biases, including social desirability and recall bias, which may have led to the overestimation of healthy behaviors across the sample [75]. Furthermore, data on adolescent’s engagement in the risk behaviors were parent reported, which limits their reliability and validity, especially in relation to substance use and sleep. Indeed, research shows that parents typically overestimate their adolescents’ sleep duration, highlighting the importance of using adolescent-derived estimates of sleep patterns in this age group [32]. Although it was not feasible to recruit parent-child dyads in this study, future research should seek to do so to supplement parent-reported data. This would provide a more comprehensive picture of health behaviors and related factors within the family unit and enable more robust explorations of whether parenting factors, such as role-modeling, rule-setting, and knowledge of guidelines, are associated with improved adolescent risk behaviors. The inclusion of peers, who become particularly influential among the older adolescents, may also be of benefit in such research.

### Conclusions

This cross-sectional survey of Australian parents revealed important findings regarding information needs and intervention preferences for preventing multiple lifestyle risk behaviors among adolescents. The results suggest that to promote healthy behaviors among adolescents, future parent-based interventions should be delivered via eHealth methods, and they must aim to increase parental knowledge of health guidelines; provide opportunities for skill-building, such as goal-setting; and include effective BCTs such as motivational interviewing and social support.

## Appendix

Multimedia Appendix 1 Additional analyses, recruitment flyer, and national health guidelines for adolescents.

## Abbreviations

BCT: behavior change technique
MVPA: moderate to vigorous physical activity
