# Supplementary material for: Parental Information Needs and Intervention Preferences for Preventing Multiple Lifestyle Risk Behaviors Among Adolescents: Cross-sectional Survey Among Parents
Source: JMIR Pediatr Parent. 2023 Apr 4;6:e42272. doi: 10.2196/42272 (PMC10131774; doi:10.2196/42272)

**Multimedia Appendix**

**Table S.1.** Australian health guidelines for adolescents

| **Risk Behaviour** | **National guideline** |
| --- | --- |
| Alcohol use | Children and people under 18 years of age should not drink alcohol^(1)^ |
| Tobacco use | No guideline |
| Screen time | No more than 2 hours of sedentary recreational screen time per day^(2)^ |
| MVPA | At least 60 minutes each day of moderate to vigorous physical activity^(2)^ |
| Fruit/vegetable intake | - Two serves of fruit per day - At least five serves of vegetables per day ^(3)^ |
| Sleep duration | - Children aged 5 to 13 years should get 9 to 11 hours of uninterrupted sleep - Young people aged 14 to 17 years should get 8 to 10 hours of uninterrupted sleep^(2)^ |

1. National Health and Medical Research Council. Australian guidelines to reduce health risks from drinking alcohol. Canberra: Commonwealth of Australia; 2020.

2. The Australian Government Department of Health. Australian 24-Hour Movement Guidelines for Children and Young People (5 to 17 years): An Integration of Physical Activity, Sedentary Behaviour, and Sleep. Canberra: Commonwealth of Australia; 2019.

3. National Health and Medical Research Council. Australian Dietary Guidelines. Canberra: NHMRC; 2013.

**Table S2**

|  | **Pearson Correlation** | **N** |
| --- | --- | --- |
| Fruit consumption | .394** | 128 |
| Veg consumption | .625** | 126 |
| Sleep duration | .386** | 132 |
| Adolescent weekday screen time / parent total screen time | .206* | 131 |
| Adolescent weekend screen time / parent total screen time | .213* | 130 |
| Adolescent MVPA / parent moderate PA | .263** | 150 |
| Adolescent MVPA / parent vigorous PA | .285** | 150 |
|  | **Phi** |  |
| Adolescent alcohol use (never used) / Parent alcohol use (never/monthly vs other) | -0.56 | 154 |
| Ever used tobacco | 1.54 | 152 |
| Ever used e-cigarettes | .290** | 152 |

**Significant at the 0.01 level (2-tailed)

**Significant at the 0.05 level (2-tailed)

**Table S3.** Chi-square tests examining parental rule-setting by adolescent age

|  | **11-15years N, %** | **15-18years**  **N, %** |  |
| --- | --- | --- | --- |
| **Alcohol use** (% sometimes, often, always) | 57, 91.9% | 47, 74.6% | *p*=0.01 |
| **Tobacco use**  (% sometimes, often, always) | 55, 85.7% | 45, 71.4% | *p*=0.016 |
| **Physical activity**  (% sometimes, often, always) | 53, 85.5% | 49, 77.8% | *p*=0.266 |
| **Screen time**  (% sometimes, often, always) | 58, 93.5% | 54, 87.1% | *p*=0.224 |
| **Diet**  (% sometimes, often, always) | 54, 87.1% | 55, 87.3% | *p*=0.973 |
| **Sleep**  (% sometimes, often, always) | 61, 100% | 60, 96.8% | *p*=0.157 |

**Table S.4.** Logistic regressions examining parental knowledge of national guidelines and adolescent adherence to guidelines

| **Parental Knowledge** | **OR** | **95% CI** | **p-value** |
| --- | --- | --- | --- |
| Alcohol use | 2.77 | .56 to 13.77 | .21 |
| MVPA | - | - | - |
| Screen time  *School days*  *Weekend days* | .25  1.16 | .08 to .74  .10 to 13.52 | **.01**  .91 |
| Diet  *Fruit intake*  *Vegetable intake* | .82  1.90 | .37 to 1.81  .30 to 11.92 | .62  .49 |
| Sleep | 3.80 | .46 to 31.09 | .21 |

*Note. Model for MVPA was unable to converge due to small cell counts. No national guideline for tobacco use. Bolding indicates significance at p<.01*

**Table S.5.** Logistic regressions examining parental rule-setting and adolescent risk behaviours

| **Parental rule setting** | **OR** | **95% CI** | **p-value** |
| --- | --- | --- | --- |
| Alcohol use | - | - | *-* |
| Tobacco use | 1.14 | .27 to 5.78 | .87 |
| Physical activity | .93 | .10 to 8.66 | .95 |
| Screen time  *School days*  *Weekend days* | .52  .18 | .39 to 3.47  .03 to 1.09 | .27  .06 |
| Diet  *Fruit intake*  *Vegetable intake* | .57  .31 | .22 to 1.49  .05 to 1.86 | .25  .20 |
| Sleep | 6.53 | .39 to 110.11 | .19 |

*Note. Model for alcohol use was unable to converge due to small cell counts.*

Recruitment flyer


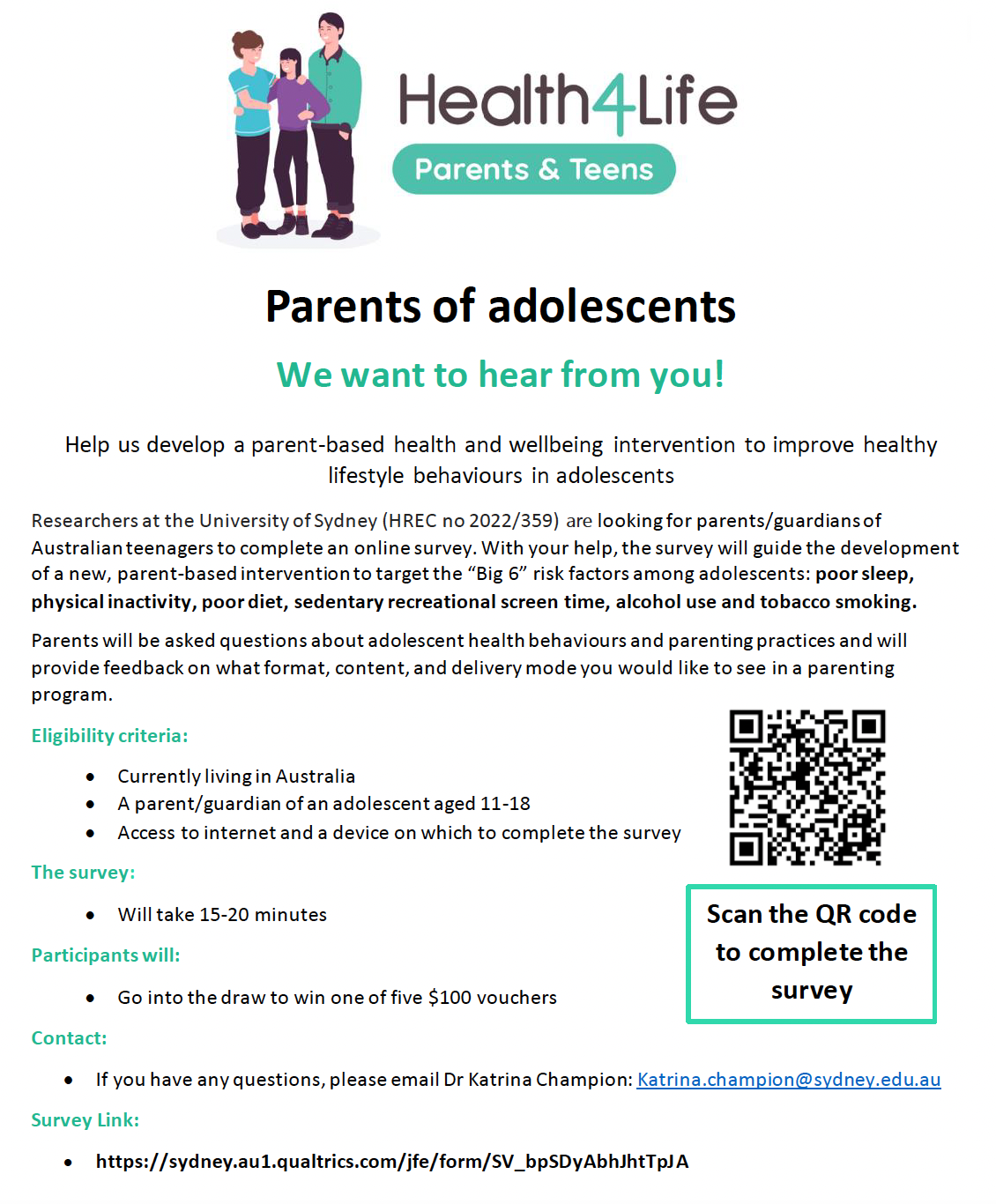

Supplement: Multimedia Appendix 1 [file pediatrics_v6i1e42272_app1.docx]
